# New ultrasound grading system for cesarean scar pregnancy and its implications for management strategies: An observational cohort study

**DOI:** 10.1371/journal.pone.0202020

**Published:** 2018-08-09

**Authors:** Shin-Yu Lin, Chia-Jung Hsieh, Yi-An Tu, Yi-Ping Li, Chien-Nan Lee, Wen-Wei Hsu, Jin-Chung Shih

**Affiliations:** 1 Department of Obstetrics and Gynecology, National Taiwan University Hospital and National Taiwan University College of Medicine, Taipei, Taiwan; 2 Department of Public Health, Tzu Chi University, Hualien, Taiwan; 3 Department of Obstetrics and Gynecology, Shin Kong Wu Ho-Su Memorial Hospital, Taipei, Taiwan; Zhejiang University School of Medicine, CHINA

## Abstract

A cesarean section pregnancy (CSP) indicated the gestational sac (GS) implanted in the previous cesarean scar. The clinical manifestations of CSP present a wide range of variations, and the optimal management is yet to be defined. We retrospectively enrolled 109 patients with the diagnosis of CSP from our department and categorized them into four grades based on the ultrasound presentation. Grade I CSP indicated the GS embedded in less than one-half thickness of the lower anterior corpus; and grade II CSP represented the GS extended to more than one-half thickness of overlying myometrium. Grade III CSP implied the GS bulged out of the cesarean scar; and grade IV CSP denoted that GS became an amorphous tumor with rich vascularity at the cesarean scar. Seventy-eight women received surgery, and the complication rate was 14.1% (11/78). Linear regression analysis demonstrated a significant association between the invasiveness of the surgery and their ultrasound gradings. The mainstream operation for grade I CSP was transcervical resection, while the majority of grade III and IV patients required hysterotomy or hysterectomy. Another 31 women received chemotherapy with methotrexate as their initial treatment. The success rate for chemotherapy was 61.3%; the remaining patients required further surgery due to persistent CSP or heavy bleeding during or after chemotherapy. Fifteen patients (48.3%) receiving chemotherapy suffered from complications (mostly bleeding). Among them, 7 (22.6%) patients experienced bleeding of more than 1,000 mL, and 9 (29.0%) of these 31 patients required blood transfusions. Our novel ultrasound grading system for CSP may help to communicate between physicians, and determine the optimal surgical strategy. Chemotherapy with methotrexate for CSP is not satisfactory and is associated with a higher rate of complications.

## Introduction

A cesarean scar pregnancy (CSP) is a rare form of ectopic pregnancy in which the gestational sac (GS) implanted inside the previous cesarean scar. The reported incidence of CSP ranges widely from 1/800 to 1/2500 [1–3]. Emerging evidence suggests that the primary cause of CSP is the damage to the endometrium and myometrium by previous cesarean section [4]. If left untreated, CSP may progress into an abnormally invasive placenta, which can result in uterine rupture and life-threatening hemorrhage [5–7]. Therefore, early recognition and timely management are essential for the optimization of therapy and improved patient outcomes.

Most of the management options for CSP are based on a single case or case series [8–11]. Generally, termination of a pregnancy in the first trimester is strongly recommended [12]. Conservative treatment with local or systemically administered methotrexate (MTX) carries a risk of heavy bleeding [13, 14]. Surgical treatment includes excision of the gestational tissues by laparoscopy [9], hysterotomy [15], or hysterectomy [16]. Other treatment choices include dilatation and curettage [17], transcervical resection (TCR) by hysteroscopy [17], uterine artery embolization (UAE), uterine artery chemoembolization [18], or, recently, placement of a double-balloon catheter [19]. Researchers have proposed various management plans based on the gestational age, embryo viability, evidence of myometrial deficiency, and clinical symptoms at presentation [20]. Unfortunately, there are no standardized guidelines for management [21].

Vial et al. classified CSP into endogenic and exogenic types [14]. The endogenic type occurs when the CSP progressed into either the cervico-isthmic region or the endometrial cavity. The exogenic type denotes the deep implantation of CSP with progression towards the overlying myometrium. Although many interventions have been suggested, there is no consensus regarding the optimal management of the endogenic and exogenic types of CSP [16]. Hwang et al. also categorized CSP into two types: intramural and non-intramural [22]. However, it was difficult to draw firm conclusions based on the 22 cases analyzed. Zhang et al. classified CSP into risky and stable types, in which the risky type was further categorized into type I (Ia, Ib, Ic), type II, and type III based on the GS location and remaining myometrial thickness [23]. This classification was shown to provide a better treatment option for different types of CSP; however, this classification is somewhat complicated for most obstetricians. In this study, we categorized patients with CSP into four grades according to their ultrasonographic findings and wish to develop a management strategy based on the ultrasound grading system.

## Materials and methods

A retrospective analysis of CSP was performed by reviewing the medical records of 109 patients with CSP who were diagnosed and treated at our hospital between 1994 and 2015. Transabdominal and transvaginal sonographic examinations were performed by HDI 3000 (Advanced Technologies and Laboratories, Bothell, WA, US) and Voluson series (GE Healthcare, Milwaukee, WI) ultrasound equipment. Demographic and clinical data were recorded, including maternal age, parity, gestational age, the presenting symptoms, the management method and the clinical outcome with the approval of our Institutional Review Board of Research Ethical Committee and our hospital authority (NTUH- 201507049RINB).

### Ultrasound grading of cesarean scar pregnancy

**Fig 1:**
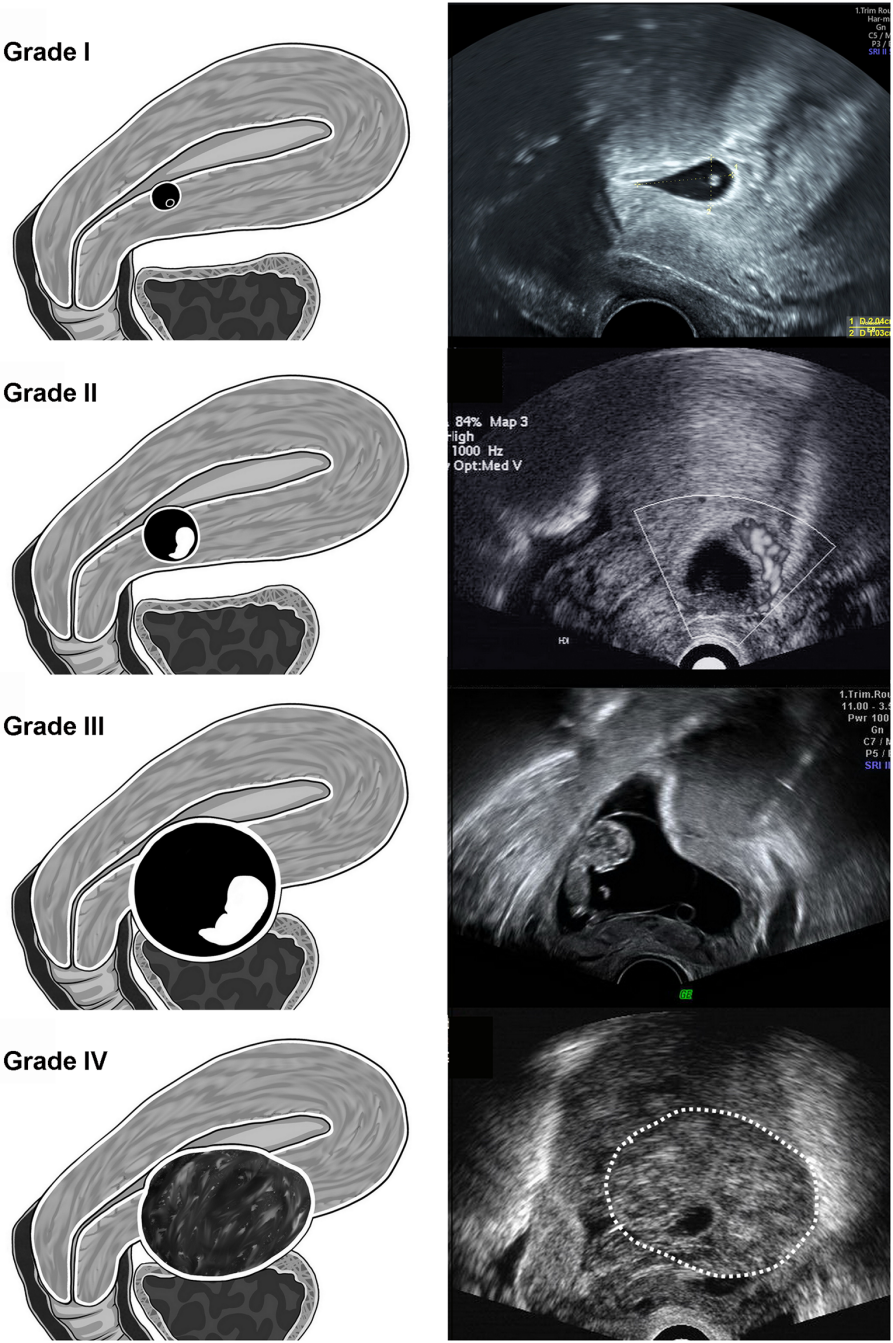
The description of our new ultrasound grading system for CSP. Grade I CSP represented the depth of CSP embedded in less than one-half thickness of the lower anterior corpus. Grade II CSP implied CSP occupied more than one-half thickness of the lower anterior corpus. In grade III CSP, the GS bulged out the overlying myometrium and uterine serosa. In grade IV CSP, the GS became an amorphous tumor with rich vascularity at the cesarean scar.

CSPs were retrospectively categorized into four grades solely based on the sonographic findings by JC Shih in 2017. We defined grade I CSP when the GS was embedded in less than one-half thickness of the myometrium. Grade II CSP denoted that CSP occupied more than one-half depth of the implanted myometrium. In grade III CSP, the GS bulged out of the overlying myometrium and serosa. Grade IV CSP indicated that the GS became an amorphous tumor with rich vascularity at the site of previous cesarean scar (Fig 1). We have tested 15 random images to two different doctors who are familiar with the definition of the ultrasound grading for CSP and blind to the clinical variables. The grading independently made by them revealed almost perfect of the reliability for inter-rater agreement on ultrasound grading (Kappa statistics coefficient = 0.81).

### Treatment options of cesarean scar pregnancy

In this cohort, women with a hemodynamically stable condition were considered suitable for all management options including medical treatment with methotrexate or surgical removal. If the patient presented with massive bleeding, surgical removal and hemostasis would be the only choice. All the patients were counseled by the available management alternatives, including the potential benefits, risks and failure rate of treatment. Unlike tubal pregnancy, CSP did not have a clear guideline of MTX treatment [24, 25]. We explained the successful rate and potential risk of MTX to our patients, yet no strict criteria that prohibit our patients to receive MTX (even with initial β-hCG > 100,000 IU/L). Written informed consent was obtained from the patients before treatment.

Systemic chemotherapy was performed with an intramuscular injection of MTX 1 mg/kg on days 1, 3, 5, and 7 and tetrahydrofolate 0.1 mg/kg on days 2, 4, 6, and 8. If the serum assay of the β-subunit of human chorionic gonadotropin (β-hCG) did not drop by 15% one week later, a second dose of MTX was administered. Local injection of MTX into the GS was also performed in some patients with a single dose of 50 mg/m^2^ body surface area (BSA). The definition of complication of chemotherapy was blood loss of more than 500 mL during and after treatment. The additional hemostatic procedures for bleeding complications included Foley catheter tamponade and UAE. The follow-up protocol for these chemotherapy patients included weekly measurement of the serum level of β-hCG for 3 consecutive weeks and bimonthly measurements thereafter until the β-hCG level had returned to undetectable.

The surgical procedures included TCR by hysteroscopy, hysterotomy by either laparoscopy or mini-laparotomy and hysterectomy. If the patients agreed to receive surgery, the type of surgery would be based on clinician’s experience with incorporating of the patients’ desire. In our institute, we do not have strict criteria for different types of surgical treatment. However, the consensus of treatment is using the “least invasive” surgery for these different CSPs. Additionally, the benefits of surgery cannot outweigh the risk of procedure itself. For instances, hysterectomy is generally not considered for a small CSP lesion; and we won’t choose transcervical resection for CSP with a very thin overlying myometrium and/or abundant blood flow due to the risk of uterine perforation and massive bleeding. Our definition of complication included blood loss more than 500 mL or internal organ injuries associated with treatment.

Patients who experienced heavy bleeding were managed with additional UAE performed by radiologists. The anterior branch of the internal iliac artery was selectively catheterized with a 4-French sized cobra-shaped catheter (Cobra; Cordis, the Netherlands) and 0.035-inch-diameter hydrophilic polymer-coated guide-wire (Radiofocus; Terumo, Japan), followed by embolization with gelfoam sponge particles. The size of the gelfoam sponge particles used in embolization was 1–2 mm. Adequate hemostasis was achieved until flow stasis at the lower uterine segment.

### Statistical analysis

Data are expressed as mean ± standard deviation (SD) and case number (n). To compare with the differences between these ultrasound gradings, we used the one-way analysis of variance (ANOVA) test or independent t test for continuous variables, and the Chi-squared test or Fisher's exact test for categorical variables. Multiple linear regression models and trend test were used to investigate the association between invasiveness of surgery and the ultrasound grading, with the adjustment of gestational age, GS size, initial β-hCG level and presence of FHB. Statistical analyses were analyzed using IBM SPSS statistical software (Version 18.0, SPSS Inc., Chicago, USA). All tests were two-tailed and with statistical significance achieved at p < 0.05.

### Details of ethic approval

We have obtained the official approving from our Institutional Review Board (NTUH- 201507049RINB) for this retrospective chart review. Although the data of all patients are not fully anonymized during access, our hospital has a strict regulation in protecting the privacy of the patients. Specifically, the patient’s data are locked. We can only access with specific identity password; and this user identity password will leave a record in the hospital domain. We are requested to keep all data confidential and strictly for the purpose for this chart review. All the subject data cannot be disclosed to either public or inside our institution. The hospital authority audits us every 6 months to ensure that we adhere to this regulation to protect patients’ privacy and security. Moreover, we also apply for the exemption from informed consent of patients due to (1) the study is at the lower risk of the patients; (2) the cohort of this chart review extended more than two decades, several patients cannot be reached using their contact information leaving in the hospital; (3) we cannot proceed the analysis if the inform consent is not exempted. The hospital authority has waived us to obtain the written consent from these patients.

## Results

According to our sonographic grading, 14, 47, 17, and 31 patients of the 109 women were categorized as grade I, grade II, grade III and grade IV, respectively. There was no statistically significant difference in the demographic characteristics between the four groups, except for the presenting gestational age (Table 1).

**Table 1:** Demographic characteristics (n = 109).

	Grade I	Grade II	Grade III	Grade IV	p-value
Case numbers	14	47	17	31	
Age* (mean± SD)	34.4± 5.2	34.7± 5.3	35.1± 5.4	33.5± 4.8	0.696
Gestational weeks* (mean± SD)	6.1± 0.6	7.2± 1.4	9.3± 1.6	9.0± 2.1	< 0.0001
Initial β-hCG level (IU/L)* (mean± SD)	20,156± 19,532	42,954± 36,934	48,359± 34,385	29,043± 37,881	0.053
Interval from prior cesarean section (months)* (mean± SD)	68.0± 36.0	64.7± 54.7	66± 55.1	49.9± 39.3	0.619
Number of prior cesarean sections† (n)					0.597
0–1	7	16	8	14	
≥2	7	31	9	17	
Number of prior dilatation and curettage† (n)					0.315
0	5	21	5	13	
1	3	5	4	10	
≥2	6	21	7	8	
Gravida^†^					
2–3	5	17	6	13	0.785
4–5	7	17	7	14	
≥6	2	13	4	4	
Operation^†^					< 0.0001
No	2	9	2	18	
TCR	10	7	1	0	
Hysterotomy via LSC	1	4	0	0	
Hysterotomy via laparotomy	1	25	11	8	
Hysterectomy	0	2	3	5	

n: number

*ANOVA test

^†^Chi-squared test

TCR: transcervical resection, LSC: laparoscopy

### Surgical treatment group

**Fig 2:**
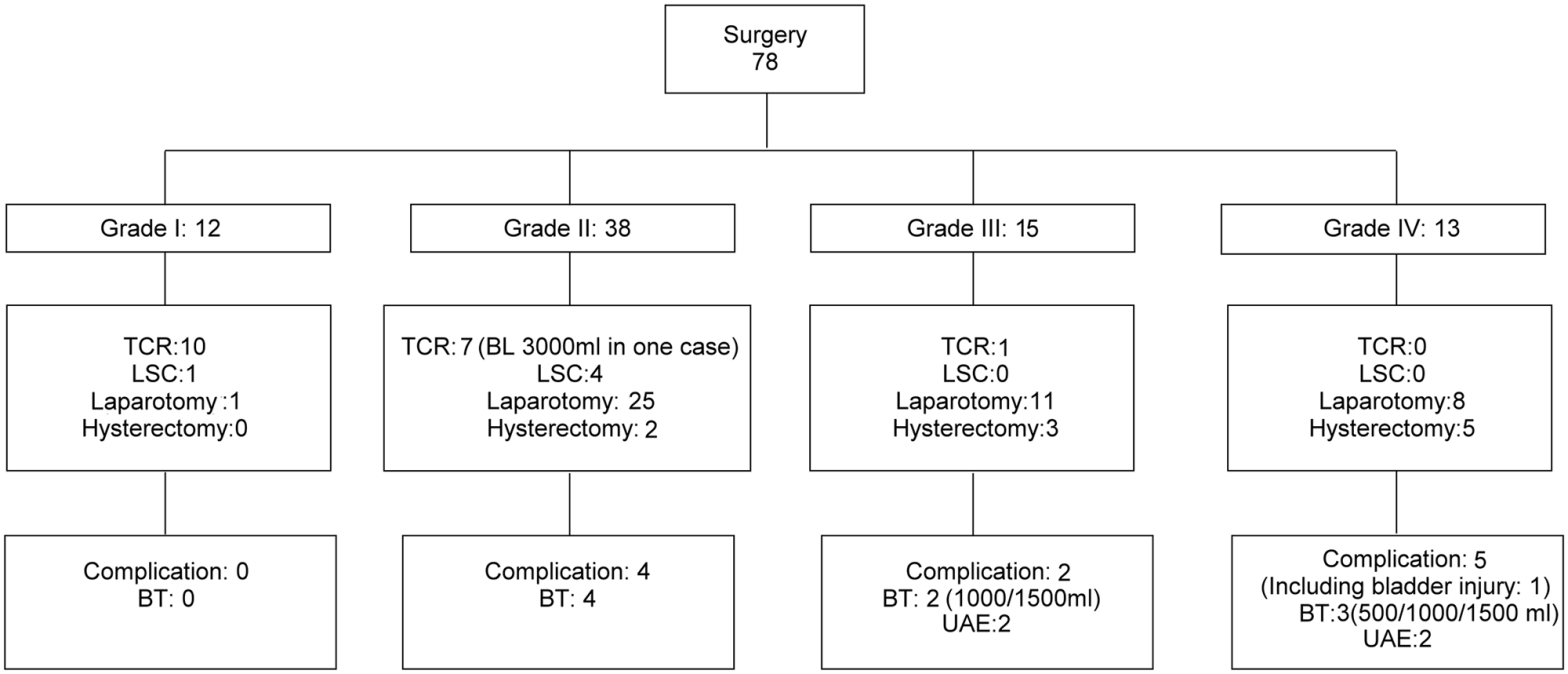
Outcome and complications in surgical patients with different grading of CSP (n = 78). TCR: Transcervical resection by hysteroscopy; LSC: Laparoscopic hysterotomy; BT: Blood transfusion; BL: Blood loss; Laparotomy: hysterotomy via mini-laparotomy or conventional laparotomy; UAE: uterine artery embolization.

Seventy-eight out of 109 (71.6%) patients received surgical intervention (Fig 2). Ten women with grade I CSP (n = 12) removed the gestational products by TCR. For those patients with grade II CSP (n = 38), seven (18.4%) patients received uterine curettage or TCR. However, two out of these patients experienced massive bleeding (1,000 and 3,000 mL, respectively), and another two patients required tamponade with a Foley catheter to stop bleeding. The majority of grade II patients (n = 29, 74.4%) received a hysterotomy either by laparoscopy (n = 4) or mini-laparotomy (n = 25). Two of these patients had excessive bleeding (500 mL and 600 mL); while no other complication was noted.

For grades III and IV, most patients underwent hysterotomy (via laparotomy, n = 19; or by hysterectomy n = 8). For grade III, two out of 11 patients underwent hysterotomy experienced major bleeding (1,500 mL and 1,000 mL) during the operation. Both patients had to receive salvage UAE for bleeding control. In one patient, we performed prophylactic UAE before hysterectomy due to its rich vascularity, but excessive bleeding more than 1,000 mL still occurred. For grade IV, two out of eight patients receiving hysterotomy encountered major bleeding (600 mL and 500 mL), and one of them needed an additional compression suture to stop the bleeding. Three out of five patients receiving hysterectomy had major bleeding (1500, 1200 and 500 mL, respectively), and two of them had to receive salvage UAE for bleeding control. One case was complicated with bladder injury during the operation.

In those patients receiving surgical treatment, excessive bleeding (more than 500 mL) occurred in 15% of patients (12/78). All patients did not require further chemotherapy for salvage treatment. Surgical complication occurred in 0, 4, 2 and 5 women in grades I, II, III, and IV, respectively. There was no statistically significant difference in the complication rate between groups (p = 0.0302 by Chi-squared test). To investigate whether the invasiveness of the surgery correlated with their initial ultrasound grading, a linear regression analysis was carried out (Table 2). The null hypothesis was that “invasiveness of surgical method” did not correlate with the “ultrasound grading”. While setting TCR as the reference, we observed a significantly higher grading score in those patients receiving hysterotomy via laparotomy (β ± SE = 1.08 ± 0.21, p < 0.0001) and hysterectomy (β ± SE = 1.80 ± 0.30, p < 0.0001). However, there was no difference of grading score between those patients receiving TCR and hysterotomy via laparoscopy groups. When the model was further adjusted with gestational age, GS size, initial β-hCG level and the presence of a fetal heartbeat (FHB), there was still a strong association between the invasiveness of the surgery and the grading score (TCR versus hysterotomy via laparotomy, β ± SE = 0.80 ± 0.20, p < 0.0001; TCR versus hysterectomy, β ± SE = 1.16 ± 0.30, p < 0.0001). Similarly, there is no significant difference between TCR and laparoscopy groups. In addition, the multivariate-adjusted analysis revealed grading score increased with the invasiveness of surgery (p for trend < 0.0001). In other words, more invasive surgery (such as hysterectomy) tended to associate with more advanced ultrasound grading.

**Table 2:** Linear regression analysis for the invasiveness of surgery and the ultrasound grading.

Operation	Number	β ± SE	p-value	P for trend
Model 1*	78			< 0.0001
TCR	18	Reference		
Hysterotomy via LSC	5	0.30 ± 0.38	0.435	
Hysterotomy via laparotomy	45	1.08 ± 0.21	< 0.0001	
Hysterectomy	10	1.80 ± 0.30	< 0.0001	
Model 2†	78			< 0.0001
TCR	18	Reference		
Hysterotomy via LSC	5	0.22 ± 0.34	0.519	
Hysterotomy via laparotomy	45	0.80 ± 0.20	< 0.0001	
Hysterectomy	10	1.16 ± 0.30	< 0.0001	

*Model 1, simple linear regression model.

^†^Model 2, multiple linear regression model adjusted for gestational age, GS size, initial β-hCG level and presence of FHB.

TCR: transcervical resection, LSC: laparoscopy

### Chemotherapy group

**Fig 3:**
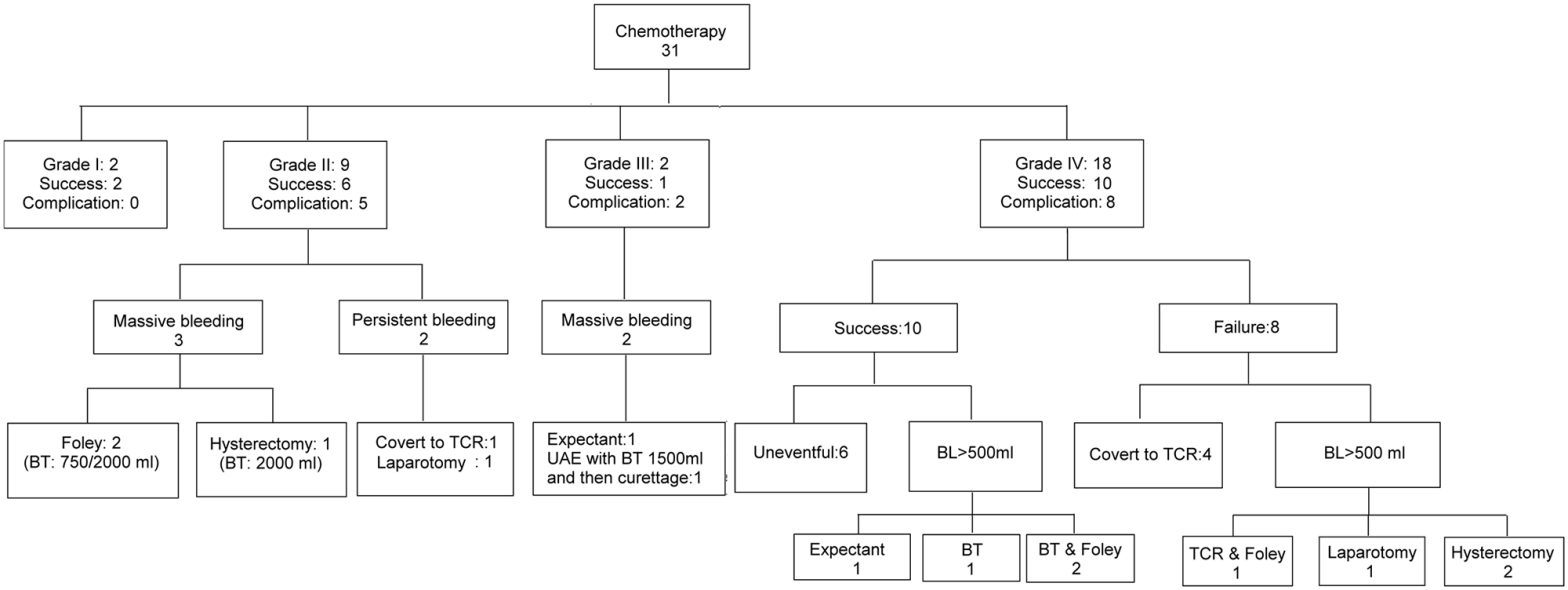
Outcome and complications of the patients receiving chemotherapy (n = 31). Foley: uterine tamponade by Foley catheter. BT: blood transfusion; TCR: transcervical resection by hysteroscopy; Laparotomy: hysterotomy via mini-laparotomy or conventional laparotomy. UAE: uterine artery embolization. BL: blood loss.

There were 31 women receiving chemotherapy as their primary treatment. The treatment results are illustrated in Fig 3. There were only 2 patients in grade I, and both of them experienced uneventful treatment. In grade II CSP (n = 9), three of the patients encountered massive vaginal bleeding during or after chemotherapy. Two of these patients required further uterine tamponade by Foley catheter, and the other patient required hysterectomy due to catastrophic bleeding (2,000 mL). In addition, another two patients with persistent vaginal bleeding had to receive TCR or hysterotomy resection to stop the bleeding and eradicate the gestational tissues.

In the two patients categorized as grade III CSP, both experienced excessive bleeding during chemotherapy (> 500 mL). One of these patients had to receive UAE followed by uterine curettage to stop bleeding and remove the gestational tissues. Among patients with grade IV CSP (n = 18), 8 experienced heavy bleeding during or after treatment. Three of these patients required uterine tamponade by Foley catheter for homeostasis. One woman received hysterotomy to stop the bleeding and CSP removal. Two patients were converted to hysterectomy. One of them was due to persistent vaginal bleeding and a gradually growing CSP after 5 courses of chemotherapy.

The total successful rate in this series was 61.3% (19/31), while the total complication rate was 48.3% (15/31). The success rate of chemotherapy was 100%, 66.7%, 50% and 55.5% in grades I, II, III, and IV, respectively, while the complication rate was 0%, 55.6%, 100% and 44.4%, respectively. Because the case numbers were limited in grades I and III, grades I-II and III-IV were merged together for further statistics due to the similarity of their ultrasound presentation. Based on Fisher’s exact test, the successful rate, complications, additional hemostatic procedures and the rate of blood transfusion showed no significant difference between grades I-II and III–IV (Table 3).

**Table 3:** The result of methotrexate treatment in different ultrasound gradings.

Grade	I-II	III-IV	p-value*
Total	11	20	
Success			0.452
No	3	9	
Yes	8	11	
Complication			0.809
No	6	10	
Yes	5	10	
Additional hemostatic procedures			0.638
No	8	17	
Yes	3	3	
Blood transfusion			0.429
No	9	13	
Yes	2	7	

* Chi-Square test or Fisher's exact test

We further applied other variables to determine whether any of them could predict the success of chemotherapy (Table 4). Except for the sac size, neither demographic characteristics (such as gestational weeks and initial β-hCG) nor ultrasound variables (presence of FHB and distance between CSP and serosa) demonstrated a good correlation with successful chemotherapy.

**Table 4:** Comparisons of demographic characteristics for successful and failed treatments of methotrexate injection.

	Success (N = 19)	Failure (N = 12)	p-value
Systemic MTX*	16‡	12	0.265
Gestational weeks† (mean ± SD)	8.11 ± 1.82	9.50±2.27	0.070
Initial β-hCG level† (mean ± SD)	39,905.0±47881.0	30,458.4±39,513.9	0.573
Sac size† (mm) (mean ± SD)	31.6 ± 13.4	41.3 ± 13.0	0.040
Positive FHB* (N)	6	5	0.705
Distance between CSP and uterine serosa† (mean± SD)	1.31 ± 2.08	2.83 ± 2.70	0.088

* Chi-Square test

^†^ Independent t test

^‡^ The other 3 patients received MTX local injection into the GS.

FHB: fetal heartbeat, CSP: cesarean scar pregnancy

Among the chemotherapy patients (n = 31), 12 (38.7%) patients were converted to surgery after chemotherapy, and six (19.4%) patients needed additional hemostatic procedures, such as Foley tamponade or UAE. Fifteen (48.4%) patients suffered from complications. Among them, 6 of 31 (19.4%) patients experienced bleeding of more than 1,000 mL, and nine (29.0%) patients required a blood transfusion.

All the necessary demographic data, treatment and outcome for these patients are supplied as S1 Table.

## Discussion

The outcome of CSP depends on a correct diagnosis and appropriate treatment. Ultrasonography provides clear image details for diagnosis, especially when performed in conjunction with appropriate clinical investigation [26]. Nevertheless, the role of ultrasound in choosing an appropriate strategy for management has seldom been described. In this retrospective study cohort, we categorized our patients with CSP into four grades according to their sonographic findings and investigated whether these ultrasound gradings correlated with the treatment methods applied and patient outcomes.

As shown in Table 1, we first demonstrated that there was no difference in the demographic characteristics of each group except for the presenting gestational weeks. Theoretically, hysterectomy can be considered for all kinds of CSP, but more invasive surgery may also bring unnecessary harm. Therefore, a least invasive approach should be considered first. However, there is no consensus of the optimal management for different types of CSP. In this cohort, TCR was the least invasive operation for CSP. We found that patients receiving hysterotomy via laparoscopy, mini-laparotomy, and hysterectomy showed a higher ultrasound grading score than the TCR group (increased by 0.22±0.34, 0.80±0.20, and 1.16±0.30, respectively). Invasive surgery for CSP was associated with a significant trend for a higher ultrasound grading (p < 0.0001). Thus, TCR is generally adequate for grade I CSP, while hysterotomy or even hysterectomy was usually required for grades III and IV CSP.

In our surgical series, most of our grade I patients received TCR for GS removal and achieved adequate hemostasis. However, only 18.4% of our grade II patients (7 of 38 women) received TCR, and two of these patients developed massive bleeding (1,000 mL and 3,000 mL; the second patient had to receive UAE and Foley compression to achieve hemostasis). The majority (n = 25, 65.8%) of the grade II patients required laparoscopic resection or hysterotomy because of the deep sac invasion and high risk of uterine perforation by TCR. In grade III, 11 of 15 patients received hysterotomy via laparotomy. Because these lesions bulge out of the uterine surface and generally show rich vascularity, laparotomic resection might be safer than laparoscopic resection.

CSP may transform into a large vascular tumor (categorized as our grade IV), typically after MTX treatment or uterine evacuation for a pre-existing CSP [27–30]. In this particular situation, catastrophic bleeding often occurs during surgical intervention, in which case UAE was usually required for hemostasis. Based on the existing literature, the exact pathogenesis for this type of vascular tumor remains unknown. The bleeding during management is typically greater than with ordinary CSPs. In this situation, either TCR or laparoscopic resection may not be appropriate for hemostasis. We chose either hysterotomy or hysterectomy for a better bleeding control. Nevertheless, additional compression sutures and UAE were still needed in two patients. Even with proper preparation, excessive bleeding and bladder injury still occurred in four patients. Therefore, we suggested that grade IV CSP should be considered as a special variety of CSP that not only unique morphology required for differential diagnosis but also prone to excessive bleeding during management.

From the data presented here, it may appropriate to say that a minimally invasive approach, such as TCR, can be offered for those patients with grade I CSP, while hysterotomy via LSC or mini-laparotomy are typically adequate for grade II CSP. For grade III CSP, hysterotomy via laparotomy or sometimes hysterectomy could be considered. For grade IV patients, the surgical approach is similar to grade III, but the risk of bleeding is higher and UAE is often required.

The use of MTX provides a choice of treatment in stable patients who wish to preserve their reproductive ability [12]. Deb *et al*. suggested that medical treatment methotrexate can be considered for those CSP with gestational age before 7 weeks, β-HCG level < 5,000 IU/L, mass diameter < 25 mm, no cardiac action of embryo, and the presence of the myometrium between the GS and bladder wall [31]. In the present series, however, the success rate was only 61%. The successful rate was similar to those reports in previous literature [9, 32]. We only disclosed GS size was different between the success and failure groups (p = 0.040) in this study (Table 4). There’s no significant difference noted between the success and failure groups in terms of gestational age, initial β-HCG, positive cardiac activity, distance to the myometrium and the use of systemic MTX in this study (Table 4). Bleeding complication occurred in approximately one-half of the patients (48%). This complication rate is higher than that in the existing literature [9, 32], probably due to the different definitions of complications. Moreover, the total treatment course for MTX was undoubtedly longer than the surgery group. We further performed a Fisher’s exact test to examine whether the ultrasound grading had an association with the chemotherapy outcomes. The results showed no difference between early and advanced grading in terms of successful treatment, complications, additional hemostatic procedures and blood transfusion. In together, although MTX could be considered as the primary treatment of CSP with regard to preserving fertility, the risks and complications associated with MTX should be appropriately counseled to the patients.

### Limitations

Our study had some limitations. First, the case number of chemotherapy was limited, and the study was not a prospective randomized cohort. Indeed it’s impractical to carry on a randomized, double-blind trial for the management of these CSPs [24]. Besides, as we treated more cases of CSP with MTX, we did encounter patients with protracted courses to achieve complete resolution and some unexpectedly massive bleeding during treatment. Because it’s not a randomized trial, we have to fairly counsel these potential risks to those new patients who had not decided their primary treatment. These information, however, would have an inevitable influence on patient’s choice. After 2002, we only have 3 cases willing to receiving methotrexate as their primary treatment. Nevertheless, we think this inevitable influence from “information provided” may not have a major impact on our conclusions. First, most of the methotrexate treatment was used in 1994–2001. We didn’t pre-select those candidates for methotrexate treatment based on either the severity or ultrasound findings in that period. Based on our data, it seems reasonable to draw the conclusion that chemotherapy with methotrexate for CSP is not satisfactory and either ultrasound grading or other clinical variables cannot effectively predict the failure treatment of MTX.

In addition, we did not have strict criteria to choose the optimal surgical methods for different CSPs. The decision left solely to the attending doctors’ judgment after incorporating patients’ desire. This inevitably resulted in bias to perform further analyses. Despite that, we have the consensus that the “least invasive” surgery is used; and the benefits of surgery cannot outweigh the risk of itself. In addition, we performed this retrospective analysis and ultrasound grading only after the ending of this observation cohort. Based on the chronological order, it seems unlikely that our conclusion (linear association of “the grading score of CSP” and their “invasiveness of surgery”) will be largely distorted by previous doctor’s judgement.

Lastly, we did not include other treatment options listed in the literature for comparison; these may include UAE alone [33], UAE combined with uterine curettage [9], chemoembolization [34], double-balloon compression [19], and transvaginal hysterotomy of CSP [35, 36]. Therefore, it is difficult to identify a preferable treatment approach for distinct ultrasound gradings.

In summary, we retrospectively reviewed 109 patients with CSP and categorized them into four grades according to their ultrasonographic findings. Our purpose is to characterize and communicate these lesions between physicians to seek an optimized protocol for management. Logistic regression analysis illustrated that greater invasiveness of the surgical procedure was associated with a higher clinical grade. In this point-of-view, our grading system did distinguish lesions that needed less invasive surgery from those CSP required for advanced surgery. The result of chemotherapy with MTX for CSP was not satisfactory compared to that of the surgery group, and neither demographic parameters nor ultrasound grading could predict successful treatment.

## Supporting information

S1 Table Demography data and the ultrasound grading in these patients with cesarean scar pregnancy. Those patients receiving methotrexate as primary therapy (within the top 31 rows) are separated with patients receiving surgical treatment using dash line.
(DOC)
