# Supplementary material for: New ultrasound grading system for cesarean scar pregnancy and its implications for management strategies: An observational cohort study
Source: PLoS One. 2018 Aug 9;13(8):e0202020. doi: 10.1371/journal.pone.0202020 (PMC6084953; doi:10.1371/journal.pone.0202020)
Supplement: S1 Table — Those patients receiving methotrexate as primary therapy (within the top 31 rows) are separated with patients receiving surgical treatment using dash line. (DOC) [file pone.0202020.s001.doc]

**Supplementary table 1: demography data and the ultrasound grading in these patients with cesarean scar pregnancy. Those patients receiving methotrexate as primary therapy (within the top 31 rows) are separated with patients receiving surgical treatment using dash line.**

| **No.^1^** | **Grade^2^** | **age** | **GA^3^ (week)** | **β-hCG**  **(IU/L)** | **Interval (month)^4^** | **# of prior CS^5^** | **G^6^** | **OP^7^** | **Sac size**  **(mm)** | **FHB^8^** | **Success for MTX^9^** | **Complications** | **Adjuvant hemostatic procedures** | **Blood transfusion** | **MTX^10^** | **Distance^11^ (mm)** |
| --- | --- | --- | --- | --- | --- | --- | --- | --- | --- | --- | --- | --- | --- | --- | --- | --- |
| 10 | 1 | 36 | 6 | 37,400 | 23 | 2 | 3 | 0 | 14 | 1 | 1 | 0 | 0 | 0 | L | 4 |
| 49 | 1 | 19 | 6 | 29,098 | 24 | 1 | 1 | 0 | 17 | 0 | 1 | 0 | 0 | 0 | S | 5 |
| 6 | 2 | 22 | 7 | 29,606 | 24 | 2 | 2 | 0 | 32 | 0 | 1 | 0 | 0 | 0 | S | 6 |
| 12 | 2 | 27 | 9 | 42,000 | 70 | 2 | 1 | 0 | 29 | 0 | 1 | 0 | 0 | 0 | S | 3 |
| 13 | 2 | 28 | 6 | 57,216 | 4 | 2 | 1 | 0 | 12 | 1 | 0 | 1 | 0 | 0 | S | 4 |
| 16 | 2 | 28 | 7 | 138,306 | 28 | 2 | 2 | 0 | 25 | 1 | 1 | 1 | 1 | 1 | L | 4 |
| 24 | 2 | 35 | 7 | 23,660 | 124 | 1 | 3 | 0 | 16 | 1 | 1 | 0 | 0 | 0 | S | 5 |
| 26 | 2 | 35 | 12 | 12,008 | 23 | 1 | 2 | 0 | 42 | 0 | 0 | 1 | 1 | 1 | S | 4 |
| 32 | 2 | 36 | 7 | 12,008 | 48 | 1 | 3 | 0 | 34 | 1 | 0 | 1 | 0 | 0 | S | 4 |
| 41 | 2 | 39 | 7 | 41,141 | 48 | 2 | 2 | 0 | 29 | 0 | 1 | 1 | 0 | 0 | S | 2 |
| 65 | 2 | 36 | 7 | 42,277 | 50 | 2 | 2 | 0 | 20 | 1 | 1 | 0 | 1 | 0 | S | 3 |
| 23 | 3 | 29 | 11 | 9,814 | 12 | 1 | 1 | 0 | 52 | 0 | 1 | 1 | 0 | 0 | S | 9 |
| 102 | 3 | 37 | 9 | 115,094 | N/A | 1 | 2 | 0 | 33 | 1 | 0 | 1 | 0 | 1 | S | 0 |
| 1 | 4 | 32 | 7 | 5,210 | 36 | 1 | 2 | 0 | 56 | 0 | 1 | 1 | 0 | 1 | S | 0 |
| 2 | 4 | 22 | 8 | 67,011 | 10 | 1 | 1 | 0 | 55 | 0 | 0 | 1 | 0 | 1 | S | 0 |
| 3 | 4 | 35 | 7 | 310 | 48 | 1 | 1 | 0 | 42 | 0 | 0 | 0 | 0 | 0 | S | 0 |
| 8 | 4 | 36 | 12 | 2,920 | 25 | 2 | 1 | 0 | 52 | 0 | 0 | 0 | 0 | 0 | S | 0 |
| 9 | 4 | 31 | 12 | 320 | 31 | 2 | 1 | 0 | 58 | 0 | 0 | 0 | 0 | 0 | S | 0 |
| 14 | 4 | 32 | 8 | 6,262 | 18 | 1 | 2 | 0 | 21 | 0 | 1 | 1 | 1 | 1 | S | 0 |
| 15 | 4 | 33 | 8 | 420 | 86 | 2 | 1 | 0 | 47 | 0 | 0 | 1 | 0 | 1 | S | 0 |
| 17 | 4 | 38 | 8 | 181,840 | 50 | 2 | 3 | 0 | 33 | 1 | 1 | 1 | 1 | 1 | S | 4 |
| 18 | 4 | 36 | 9 | 19,940 | 105 | 1 | 1 | 0 | 38 | 0 | 1 | 0 | 0 | 0 | S | 0 |
| 19 | 4 | 33 | 7 | 87 | 24 | 2 | 2 | 0 | 34 | 0 | 1 | 1 | 0 | 1 | S | 0 |
| 21 | 4 | 28 | 12 | 12,296 | 29 | 1 | 1 | 0 | 33 | 0 | 0 | 0 | 0 | 0 | S | 0 |
| 25 | 4 | 32 | 12 | 638 | 11 | 2 | 1 | 0 | 49 | 0 | 1 | 0 | 0 | 0 | L | 0 |
| 27 | 4 | 37 | 10 | 3,206 | 26 | 1 | 2 | 0 | 42 | 1 | 0 | 0 | 0 | 0 | S | 0 |
| 28 | 4 | 35 | 12 | 73,674 | 14 | 2 | 1 | 0 | 27 | 0 | 1 | 1 | 0 | 0 | S | 0 |
| 30 | 4 | 29 | 11 | 82,692 | 36 | 1 | 2 | 0 | 46 | 1 | 0 | 1 | 1 | 0 | S | 0 |
| 31 | 4 | 38 | 8 | 1,102 | 36 | 2 | 2 | 0 | 40 | 0 | 1 | 0 | 0 | 0 | S | 0 |
| 40 | 4 | 39 | 7 | 65,585 | 60 | 1 | 2 | 0 | 25 | 1 | 1 | 0 | 0 | 0 | S | 2 |
| 95 | 4 | 22 | 9 | 10,556 | N/A | 1 | 1 | 0 | 43 | 0 | 1 | 0 | 0 | 0 | S | 0 |
| 59 | 1 | 37 | 7 | 68,900 | 69 | 2 | 2 | 1 | 12 | 1 |  |  |  |  |  |  |
| 61 | 1 | 33 | 6 | 14,024 | 84 | 1 | 2 | 1 | 6 | 1 |  |  |  |  |  |  |
| 64 | 1 | 35 | 7 | 24,394 | 96 | 2 | 3 | 1 | 19 | 1 |  |  |  |  |  |  |
| 68 | 1 | 37 | 6 | 21 | 120 | 2 | 2 | 1 | 10 | 0 |  |  |  |  |  |  |
| 86 | 1 | 30 | 7 | 2,651 | N/A | 1 | 2 | 1 | 8 | 1 |  |  |  |  |  |  |
| 92 | 1 | 32 | 6 | 3,320 | N/A | 2 | 1 | 1 | 7 | 1 |  |  |  |  |  |  |
| 94 | 1 | 34 | 5 | 10,057 | N/A | 2 | 1 | 1 | 10 | 1 |  |  |  |  |  |  |
| 103 | 1 | 38 | 6 | 3,903 | N/A | 1 | 2 | 1 | 6 | 0 |  |  |  |  |  |  |
| 106 | 1 | 37 | 7 | 18,054 | N/A | 2 | 2 | 1 | 20 | 1 |  |  |  |  |  |  |
| 109 | 1 | 40 | 5 | 9,721 | N/A | 1 | 1 | 1 | 17 | 0 |  |  |  |  |  |  |
| 39 | 2 | 43 | 7 | 75,908 | 270 | 2 | 2 | 1 | 22 | 0 |  |  |  |  |  |  |
| 63 | 2 | 28 | 6 | 92,928 | 7 | 1 | 1 | 1 | 25 | 1 |  |  |  |  |  |  |
| 82 | 2 | 31 | 6 | 55,854 | N/A | 2 | 1 | 1 | 30 | 0 |  |  |  |  |  |  |
| 87 | 2 | 42 | 8 | 13,408 | N/A | 2 | 1 | 1 | 20 | 0 |  |  |  |  |  |  |
| 89 | 2 | 46 | 7 | 11,511 | N/A | 2 | 3 | 1 | 13 | 1 |  |  |  |  |  |  |
| 96 | 2 | 30 | 5 | 8,071 | N/A | 1 | 1 | 1 | 20 | 0 |  |  |  |  |  |  |
| 108 | 2 | 39 | 10 | 98,745 | N/A | 2 | 3 | 1 | 24 | 1 |  |  |  |  |  |  |
| 58 | 3 | 37 | 11 | 48,512 | 132 | 1 | 1 | 1 | 67 | 1 |  |  |  |  |  |  |
| 43 | 1 | 37 | 6 | 15,002 | 60 | 1 | 2 | 2 | 10 | 0 |  |  |  |  |  |  |
| 47 | 2 | 42 | 9 | 36,839 | 24 | 1 | 2 | 2 | 26 | 0 |  |  |  |  |  |  |
| 53 | 2 | 34 | 7 | 6,091 | 48 | 2 | 2 | 2 | 6 | 0 |  |  |  |  |  |  |
| 54 | 2 | 40 | 6 | 30,963 | 96 | 2 | 3 | 2 | 15 | 0 |  |  |  |  |  |  |
| 60 | 2 | 43 | 7 | 26,157 | 132 | 2 | 3 | 2 | 21 | 1 |  |  |  |  |  |  |
| 112 | 1 | 37 | 6 | 45,644 | N/A | 1 | 1 | 3 | 12 | 1 |  |  |  |  |  |  |
| 33 | 2 | 30 | 7 | 13,245 | 36 | 2 | 3 | 3 | 43 | 0 |  |  |  |  |  |  |
| 34 | 2 | 37 | 7 | 14,420 | 60 | 2 | 3 | 3 | 33 | 1 |  |  |  |  |  |  |
| 36 | 2 | 31 | 8 | 12,880 | 120 | 2 | 2 | 3 | 33 | 1 |  |  |  |  |  |  |
| 37 | 2 | 34 | 6 | 56,320 | 72 | 2 | 3 | 3 | 31 | 1 |  |  |  |  |  |  |
| 42 | 2 | 37 | 6 | 8,155 | 96 | 2 | 2 | 3 | 15 | 1 |  |  |  |  |  |  |
| 44 | 2 | 33 | 6 | 8,039 | 5 | 2 | 3 | 3 | 12 | 0 |  |  |  |  |  |  |
| 46 | 2 | 29 | 8 | 36,412 | 36 | 1 | 1 | 3 | 21 | 1 |  |  |  |  |  |  |
| 50 | 2 | 27 | 6 | 41,200 | 36 | 2 | 1 | 3 | 14 | 0 |  |  |  |  |  |  |
| 52 | 2 | 42 | 12 | 41,001 | 96 | 1 | 2 | 3 | 45 | 0 |  |  |  |  |  |  |
| 57 | 2 | 34 | 7 | 44,321 | 144 | 2 | 3 | 3 | 50 | 1 |  |  |  |  |  |  |
| 66 | 2 | 35 | 6 | 25,777 | 84 | 2 | 2 | 3 | 28 | 1 |  |  |  |  |  |  |
| 69 | 2 | 36 | 7 | 67,915 | 48 | 2 | 2 | 3 | 37 | 0 |  |  |  |  |  |  |
| 71 | 2 | 39 | 9 | 183,664 | 96 | 1 | 1 | 3 | 63 | 0 |  |  |  |  |  |  |
| 72 | 2 | 39 | 7 | 100,459 | 46 | 2 | 2 | 3 | 28 | 1 |  |  |  |  |  |  |
| 74 | 2 | 32 | 7 | 56,330 | N/A | 2 | 1 | 3 | 26 | 1 |  |  |  |  |  |  |
| 79 | 2 | 34 | 8 | 39,502 | N/A | 2 | 2 | 3 | 17 | 1 |  |  |  |  |  |  |
| 80 | 2 | 36 | 7 | 36,521 | N/A | 1 | 1 | 3 | 22 | 1 |  |  |  |  |  |  |
| 81 | 2 | 36 | 6 | 16,563 | N/A | 1 | 1 | 3 | 18 | 1 |  |  |  |  |  |  |
| 83 | 2 | 41 | 6 | 8,397 | N/A | 2 | 1 | 3 | 9 | 0 |  |  |  |  |  |  |
| 88 | 2 | 31 | 7 | 15,016 | N/A | 2 | 1 | 3 | 12 | 0 |  |  |  |  |  |  |
| 97 | 2 | 25 | 7 | 3,025 | N/A | 1 | 2 | 3 | 23 | 1 |  |  |  |  |  |  |
| 107 | 2 | 36 | 8 | 106,041 | N/A | 1 | 1 | 3 | 21 | 1 |  |  |  |  |  |  |
| 110 | 2 | 31 | 8 | 21,415 | N/A | 1 | 3 | 3 | 18 | 1 |  |  |  |  |  |  |
| 111 | 2 | 37 | 7 | 34,607 | N/A | 1 | 1 | 3 | 10 | 0 |  |  |  |  |  |  |
| 113 | 2 | 35 | 7 | 58,054 | N/A | 2 | 3 | 3 | 24 | 1 |  |  |  |  |  |  |
| 51 | 3 | 39 | 11 | 95,600 | 24 | 2 | 2 | 3 | 67 | 1 |  |  |  |  |  |  |
| 55 | 3 | 43 | 11 | 23,559 | 72 | 2 | 1 | 3 | 54 | 1 |  |  |  |  |  |  |
| 56 | 3 | 38 | 11 | 14,400 | 24 | 2 | 2 | 3 | 19 | 0 |  |  |  |  |  |  |
| 67 | 3 | 38 | 8 | 108,858 | N/A | 2 | 3 | 3 | 48 | 1 |  |  |  |  |  |  |
| 77 | 3 | 41 | 11 | 48,412 | N/A | 1 | 1 | 3 | 76 | 1 |  |  |  |  |  |  |
| 84 | 3 | 36 | 9 | 38,466 | N/A | 1 | 1 | 3 | 22 | 1 |  |  |  |  |  |  |
| 90 | 3 | 32 | 8 | 48,656 | N/A | 2 | 3 | 3 | 42 | 1 |  |  |  |  |  |  |
| 91 | 3 | 32 | 7 | 33,744 | N/A | 2 | 2 | 3 | 20 | 1 |  |  |  |  |  |  |
| 93 | 3 | 25 | 7 | 26,400 | N/A | 1 | 1 | 3 | 33 | 1 |  |  |  |  |  |  |
| 100 | 3 |  | 8 | 22,451 | N/A | 1 | 2 | 3 | 43 | 1 |  |  |  |  |  |  |
| 104 | 3 | 32 | 7 | 44,566 | N/A | 2 | 2 | 3 | 33 | 0 |  |  |  |  |  |  |
| 22 | 4 | 34 | 8 | 9,560 | 48 | 1 | 1 | 3 | 25 | 0 |  |  |  |  |  |  |
| 35 | 4 | 25 | 6 | 67,110 | 19 | 2 | 2 | 3 | 46 | 0 |  |  |  |  |  |  |
| 38 | 4 | 36 | 10 | 23,139 | 60 | 2 | 2 | 3 | 34 | 0 |  |  |  |  |  |  |
| 45 | 4 | 35 | 15 | 22,658 | 60 | 2 | 2 | 3 | 52 | 0 |  |  |  |  |  |  |
| 48 | 4 | 35 | 7 | 32,794 | 108 | 1 | 3 | 3 | 17 | 0 |  |  |  |  |  |  |
| 62 | 4 | 27 | 7 | 849 | 72 | 2 | 3 | 3 | 28 | 1 |  |  |  |  |  |  |
| 78 | 4 | 38 | 8 | 34,518 | N/A | 1 | 1 | 3 | 43 | 0 |  |  |  |  |  |  |
| 98 | 4 | 34 | 9 | 5,312 | N/A | 2 | 2 | 3 | 30 | 0 |  |  |  |  |  |  |
| 11 | 2 | 38 | 6 | 69,236 | 10 | 1 | 1 | 4 | 32 | 1 |  |  |  |  |  |  |
| 20 | 2 | 31 | 8 | 45,670 | 24 | 2 | 2 | 4 | 20 | 0 |  |  |  |  |  |  |
| 70 | 3 | 36 | 9 | 26,338 | 132 | 2 | 3 | 4 | 51 | 0 |  |  |  |  |  |  |
| 73 | 3 | 25 | 9 | 18,983 | N/A | 2 | 3 | 4 | 35 | 0 |  |  |  |  |  |  |
| 99 | 3 | 41 | 11 | 98,265 | N/A | 1 | 2 | 4 | 58 | 0 |  |  |  |  |  |  |
| 4 | 4 | 30 | 7 | 36,752 | 7 | 2 | 2 | 4 | 54 | 0 |  |  |  |  |  |  |
| 5 | 4 | 38 | 9 | 48,464 | 96 | 2 | 3 | 4 | 27 | 1 |  |  |  |  |  |  |
| 7 | 4 | 40 | 8 | 36,659 | 48 | 2 | 2 | 4 | 50 | 0 |  |  |  |  |  |  |
| 29 | 4 | 40 | 9 | 33,450 | 184 | 1 | 2 | 4 | 55 | 0 |  |  |  |  |  |  |
| 101 | 4 | 37 | 9 | 15,000 | N/A | 2 | 1 | 4 | 41 | 0 |  |  |  |  |  |  |

**^1^ No. : Patient’s sequence number according to the date of admission; ^2^ Grade: ultrasound grading. 1 = I; 2 = II, 3 = III, 4 = IV; ^3^ GA: Gestational age at diagnosis; ^4^ Interval: Interval between the CSP and last cesarean section; ^5^ # of prior CS: Number of previous cesarean section; ^6^ G: Gravida; ^7^ OP: Operation type-- 0: no operation; 1: Transcervical resection; 2: Laparoscopic hysterotomy 3. Hysterotomy via laparotomy; 4.Hysterectomy; ^8^ FHB: Fetal heart beats. ^9^ MTX: methotrexate; ^10^ MTX: Methotraxate treatment: L: local injection; S: systemic injection; ^11^ Distance: distance between the gestational sac and overlying uterine serosa (mm). N/A: not available. In columns under the title of “Success for MTX”, “Complications”, “Adjuvant hemostastic procedures”, “Blood transfusion”, “0” stands for absent and “1” stands for present.**
